# Effects of stable ectopic expression of the primary sex determination gene *Yob* in the mosquito *Anopheles gambiae*

**DOI:** 10.1186/s13071-018-3211-z

**Published:** 2018-12-24

**Authors:** Elzbieta Krzywinska, Jaroslaw Krzywinski

**Affiliations:** Vector Molecular Biology Group, The Pirbright Institute, Pirbright, UK

**Keywords:** malaria vector, genetic sexing, transgenic mosquitoes, development

## Abstract

**Background:**

Mosquito-borne diseases, such as malaria, are controlled primarily by suppressing mosquito vector populations using insecticides. The current control programmes are seriously threatened by the emergence and rapid spread of resistance to approved insecticides. Genetic approaches proposed to complement the existing control efforts may be a more sustainable solution to mosquito control. All such approaches would rely on releases of modified male mosquitoes, because released females would contribute to biting and pathogen transmission. However, no sufficiently large-scale methods for sex separation in mosquitoes exist.

**Results:**

Here we exploited the female embryo-killing property of the sex determining gene *Yob* from the African malaria mosquito, *Anopheles gambiae*, to evaluate the feasibility of creating transgenic *An. gambiae* sexing strains with a male-only phenotype. We generated *An. gambiae* lines with *Yob* expression, in both sexes, controlled by the *vas2* promoter. Penetrance of the female-lethal phenotype was highly dependent on the location of the transgenic construct within the genome. A strong male bias was observed in one of the lines. All the females that survived to adulthood in that line possessed masculinized head appendages and terminal abdominal segments. They did not feed on blood, lacked host-seeking behavior, and thus were effectively sterile. Males, however, were not affected by *Yob* overexpression.

**Conclusions:**

Our study demonstrates that ectopic expression of *Yob* results in a recovery of viable, fertile males, and in death, or otherwise strongly deleterious effects, in females. This result shows potential for generation of transgenic sexing strains of *Anopheles gambiae* with a conditional male-only phenotype.

## Background

In many animals with chromosomal sex determination, females are homogametic and have a pair of X chromosomes, whereas males are heterogametic and possess the X and the Y chromosome in their karyotypes [1]. In the great majority of these organisms, the Y chromosome harbours a primary signal gene, often referred to as the M factor, which is actively involved in the male sex determination.

The endeavours to reveal the molecular identity of the M factor in insects proved successful only recently. These pioneering studies focused on three dipteran species and revealed a different origin of the M factor in each case [2–4], reflecting an acknowledged high diversity of the insect primary sex determination genes in general. Genes located downstream from the primary signal are much more evolutionarily constrained, and in all known instances are represented by *transformer* (*tra*) and *doublesex* (*dsx*). Both the *tra* and *dsx* genes are sex-specifically regulated at the RNA-splicing level. A common pattern emerging from observations in non-drosophilid holometabolan insects is that the female variant of *tra* encodes a functional protein that serves as a splice regulator of *dsx* and of *tra* itself. The *tra* self-splicing regulatory feedback loop is initiated in female embryos by maternally supplied TRA. In male embryos the loop is believed to be interrupted by the activity of M. The *tra* pre-mRNA is then spliced by default into a non-productive form and, consequently, in the absence of TRA, *dsx* is spliced into a male-specific form, also by default. The *dsx* gene constitutes the terminal step in the pathway and its sex-specific transcripts encode male and female forms of a transcription factor that controls all downstream sexual differentiation processes.

The sex determination pathway in mosquitoes is poorly understood. In the African malaria mosquito *Anopheles gambiae*, *dsx* [5] and, more recently, the Y chromosome-linked primary signal gene *Yob* that confers maleness [3] have been described. Molecular interactions involving these two genes remain unknown. In male embryos, *Yob* is activated shortly after oviposition, at the beginning of zygotic transcription. The sex-specific splicing of *dsx* is established several hours later. Involvement of *Yob* in the sex determination pathway and its relation to *dsx* has been demonstrated in transfection experiments, in which introduction of the *Yob* mRNA into the female *An. gambiae* cells led to a shift in *dsx* splicing from the female form towards the male form. These results show that *Yob* alone is sufficient to control splicing of *dsx* in the female cellular environment*.* Microinjection of the *Yob* mRNA into unsexed *An. gambiae* and *An. arabiensis* pre-blastoderm embryos revealed an unexpected property of *Yob*: ectopic delivery of its mRNA leaves males unaffected, but causes death of female embryos. The indiscriminate female lethality is most likely caused by misregulation of dosage compensation (the process that naturally drives hyper-activation of genes from the single X chromosome in *An. gambiae* males to the levels of expression from the two X chromosomes in females [6]). Credence to that notion is given by the experiments on transient *Yob* knockdown, which results in male embryo killing. These opposite lethal effects indicate that, indeed, *Yob* regulates dosage compensation. Consequently, presence of *Yob* mRNA in female embryos likely activates the dosage compensation complex and causes gene overexpression from both X chromosomes, leading presumably to an accumulation of toxic levels of the X chromosome transcripts. Depletion of the *Yob* transcript levels, in turn, prevents activation of the dosage compensation complex, and leads to death of males due to an insufficient transcription from the single X. Similar single-sex lethal effects in response to improper gene expression have been attributed to aberrant activation of dosage compensation in insects as diverse as *Drosophila* [7], a beetle *Tribolium castaneum* [8], and a lepidopteran *Bombyx mori* [9].

*Anopheles gambiae* is the most important vector of human malaria in sub-Saharan Africa. Control of the disease relies heavily on the use of pyrethroid impregnated bednets and on indoor residual spraying with insecticides to control the vectors. Widespread use of bednets by the local communities, in particular, proved highly effective and largely contributed to a reduction of the annual malaria mortality rates by half in the last decade. However, the side effect of the scale-up of these control operations is the strong selection pressure on mosquitoes and concomitant emergence and rapid increase in the insecticide resistance in many mosquito populations [10]. These deeply worrying developments threaten the recent gains against malaria and the overall success of ongoing control programmes.

Genetic control methods aiming at suppression of mosquito numbers, or modification of vector populations to decrease their vector competence, have been proposed as a complement to the existing control efforts. All the proposed programmes rely on release of modified mosquitoes and must incorporate male-only releases, because released females would contribute to biting and parasite transmission. However, no large-scale methods for sexing of *An. gambiae* exist. The female embryo-killing property of *Yob* offers an excellent tool to create transgenic *An. gambiae* sexing strains, which would allow efficient mass production of male-only generations for field releases in various genetic control programmes. In this study, which we regard as an initial step towards this direction, we have generated the first transgenic *An. gambiae* strains ectopically expressing *Yob* in both sexes. The construct used for transgenesis contained *Yob* cDNA under the control of *vas2* promoter. *vas2* has been originally described as an *An. gambiae* promoter with germline-specific activity [11]. However, in certain *An. gambiae* transgenic lines it is active in somatic tissues throughout the body [12]. Therefore, we exploited here the leakiness of *vas2* promoter and its presumed weak activity in somatic cells*.* The choice of *vas2* was motivated by a concern that expression of *Yob* transgene driven by strong promoters, apart from the expected lethal effect on females, may be deleterious or lethal to males, and prevent recovery of transgenic individuals or establishing transgenic lines. Activity of an autosomally inserted *vas2*:*Yob* construct was strongly influenced by the chromosomal position effect. Whereas in all transgenic lines males were viable and fertile, in one line in particular, female development and viability was profoundly affected. Although the female-lethal phenotype was not fully penetrant, our results show potential for generation of male-only strains when *Yob* ectopic expression is driven by more suitable promoters active in early embryos.

## Methods

### Mosquito maintenance

*Anopheles gambiae* wild type mosquitoes (G3 strain) and *A. gambiae* transgenic mosquitoes were reared at 28°C and 80% humidity, according to the standard protocol [13]. The adults were provided 10% sucrose solution *ad libitum.* Human red blood cells and plasma, sourced from a blood bank, were mixed at 1:1 ratio and used for female feeding using the Hemotek membrane feeder.

### Plasmid construction and mosquito transgenesis

The *Yob* cDNA [3] cloned into a pGEM T-easy vector was used as a template to PCR-amplify the *Yob* coding sequence with primers Yob_NheI_5 (5'-TTT GGC TAG CAT GTT TGT TTT GTA TGT GTC-3') and Yob_PacI_3 (5'-TGG TTA ATT AAG ATA TTT TAA TTG TTT TTA TTC GAG CGG-3'). The PCR product was cut with NheI and PacI. The p165 vector [12] was digested with PacI and PmlI to create a vector backbone. A 0.2 kb long 3' end fragment of the *vas2* promoter, removed from the vector backbone during digestion with PmlI, was PCR-amplified using primers vasP_PmlI_F (5'-ATA TTT CGA CCA TCT TTC TTT CTC TCT CTC CAC G-3') and vas5_Nhe (5'-AAA CAT GCT AGC TTT CCT TTC TTT ATT CAC CGG A-3'), and cut with PmlI and NheI. The resulting 0.2 kb fragment and the *Yob* coding sequence were cloned into the vector backbone in a single ligation reaction to create pBac_vasYob plasmid. Identity of the inserts was confirmed by sequencing candidate clones using ABI BigDye terminator chemistry (PE Applied Biosystems) on an ABI 3130xl Genetic Analyzer.

Early preblastoderm embryos were injected with a solution containing pBac_vasYob (0.4 μg/μl) and a helper plasmid pENTR R4-vas2-Transposase-R3 (0.2 μg/μl), containing piggy-Bac transposase reading frame under the control of the *vas2* regulatory sequences [14], following previously described methods [15].

### Identification of the transgene insertion site

The integration site of the piggyBac element within the genome of line 23 mosquitoes has been identified using splinkerette PCR method according to the published protocol [16]. Genomic DNA isolated from individual pupae was used for the splinkerette PCR experiment. Amplified products containing genomic sequences flanking the 5' and 3' ends of the element were sequenced directly by Sanger sequencing using ABI BigDye terminator chemistry (PE Applied Biosystems) on an ABI 3130xl Genetic Analyzer.

### RT-PCR

RNA from whole individual mosquitoes or body parts was extracted using PureLink Mini kit (Invitrogen) according to the manufacturer's protocol. Transcription levels of *Yob* were evaluated by RT-PCR using primers T7YobF (5'-TAA TAC GAC TCA CTA TAG GGA TGT TTG TTT TGT ATG TGT CG-3') and Yob_endR (5'-GAT ATT TTA ATT GTT TTT ATT CGA GCG G-3'), and the SuperScript® III One-Step RT-PCR System with Platinum® Taq DNA Polymerase (Invitrogen) according to the manufacturer’s guidelines. RT-PCR, as described above, was also used to examine splicing patterns of the *dsx* using primers dsxF2 (5'-CCA GAA CCT GTA AAT CTC CTA C-3') and dsxR5m (5'-GAT GAC TTC ACC ACC GCT TC-3'), and of *fruitless* using primers Aga_fruF (5'-GGC AGC ATC GGA CTT GTT CA-3') and Aga_fruR (5'-ACG TCG CAC AGT TTC TCA TC-3'). A fragment of the ribosomal protein S7 gene mRNA was amplified using primers S7F (5'-TGC TGC AAA CTT CGG CTA T-3') and S7R (5'-CGC TAT GGT GTT CGG TTC C-3'), to serve as an internal control of equal sample loading in each RT-PCR experiment. Identity of the RT-PCR products with the target sequences was confirmed by sequencing.

### Microscopy

Mosquitoes were screened for the presence of DsRed marker using a Leica M165 FC microscope equipped with a DsRed filter. Images were captured with a Leica DFC365 FX camera mounted on a Leica M165 FC microscope.

### Feeding and attraction experiments

Approximately 100 transgenic female pupae from line 23 were put in a cage (BugDorm-1) with approximately 100 G3 male pupae. Emerged adults were provided with 10% sucrose solution *ad libitum.* After two days, to allow mating, females were offered a bloodmeal from a Hemotek feeder. None of the females were attracted to the feeder, unlike the wild-type females of the same age that were kept in a control cage set up in parallel with the experimental cage. Then, two- and three-day old line 23 females were tested for their attraction to a human by resting an exposed forearm above the cage (in close proximity, but no direct contact with the mosquitoes, to prevent potential biting) for 5 min and observing the females’ reaction. Following the experiments, a sample of 20 females from both experimental and control cages were dissected and their spermathecae examined for the presence of sperm. The above experiments were repeated twice.

## Results

### Generation of transgenic lines

Approximately 600 preblastoderm embryos were injected with a mixture of a helper plasmid and the pBac_vasYob plasmid, the latter including the *Yob* gene under the control of the *vasa2* promoter, and a 3xP3-DsRed fluorescent marker. Hatched G_0_ larvae (*n* = 152; 25%) were screened for transient DsRed expression, and 87 individuals (57%) with the fluorochrome visible in ventral abdominal ganglia and in anal papillae were retained for further rearing. The surviving adults were placed in 8 same-sex pools for crosses with the G3 strain wild type mosquitoes. The resulting G_1_ progeny, obtained from two consecutive gonotrophic cycles, were screened at the second larval stage for the presence of the DsRed marker in both eyes. After pupation, individuals originating from different pools and/or possessing distinct DsRed expression pattern, indicative of transgene insertions in different genomic regions, were selected to establish individual lines. Only G_1_ males were used, because previous transient *Yob* mRNA assays resulted in death of females embryos, which suggested that it may not be possible to recover transgenic lines through female founders. Crosses between the G_1_ males and the wild-type females yielded seven G_2_ families, from which four lines were selected for subsequent analyses. In each family no more than 50% of individuals inherited the DsRed marker. The transgenic lines were subsequently maintained by continuous back-crossing of the DsRed-positive males with the G3 strain females.

### Characterization of lines

**Fig. 1:**
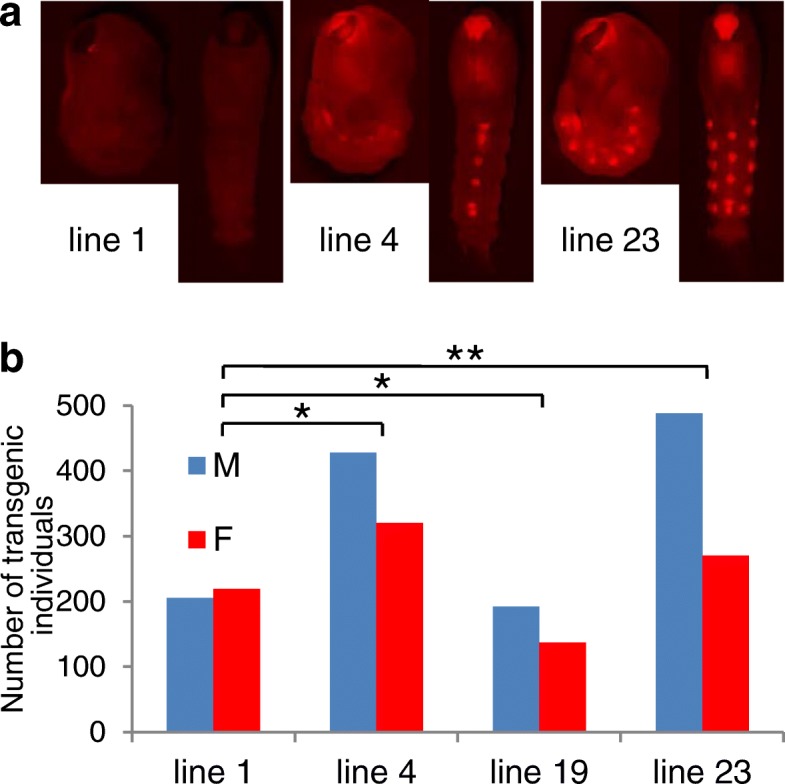
Phenotypes of the transgenic *An. gambiae* lines generated in the study. DsRed expression patterns (**a**), proportions of the sexes among transgenic individuals (**b**). Asterisks denote significant male bias: * *P* < 0.01, ** *P <* 0.0001; Chi-square test

The lines exhibited three different DsRed phenotypes (Fig. 1a). In line 1, DsRed expression was very weak and the marker was visible only in the stemmata (lateral ocelli) and optic nerves. In lines 4 and 19 (derived from two different G_0_ pools), DsRed was moderately expressed in the eyes, brain and ventral ganglia, a pattern typically observed with 3xP3-regulated expression in mosquitoes. Finally, line 23 had a strong expression of the marker in the eyes, central nervous system, and the lateral structures in the abdominal segments that seem to correspond to neurohemal organs (similar expression pattern was observed in several other transgenic lines generated in our laboratory using various piggyBac-based transgenic constructs with the 3xP3 promoter-driven expression of the marker).

Transgenic females were recovered in each line, contrary to the expectation that ectopic *Yob* expression would be female-lethal. Proportions of the sexes among transgenic individuals reaching the pupal stage differed depending on the line (Fig. 1b). Whereas in line 1 the sex ratios were approximately equal, there was a significant deficiency of female pupae in three other lines (line 4, 41.6%; line 19, 42.8%; line 23, 35.6%). Line 23 was retained for further characterization, because of its strongest female lethal effect. It has been maintained in the laboratory for over 50 generations.

### Abnormal development of line 23 females

Like other transgenic lines, line 23 was maintained through crossing of wild-type females with males heterozygous for the transgene, yielding both transgenic and wild-type progeny. Therefore, the developmental time of the transgenic and the wild-type progeny individuals could be easily compared. There was no difference in male development. However, in the transgenic females the larval stage lasted, on average, one to three days longer than in the wild-type females.

The male bias observed in pupae was caused by female mortality during the embryo stage, because no significant mortality of larvae was observed. In each generation, up to 30% of the transgenic females that survived to the pupal stage died as pupae, or during the eclosion. Moreover, among the emerged transgenic females, up to 30% were unable to fly and died shortly after the eclosion. Thus, the male bias becomes substantially more pronounced in the adult stage. The surviving females had decreased adult life span, lasting in most cases up to 7 days. None of them fed on blood, were attracted to a human arm, or mated, as indicated by a lack of sperm in the spermatheca.

**Fig. 2:**
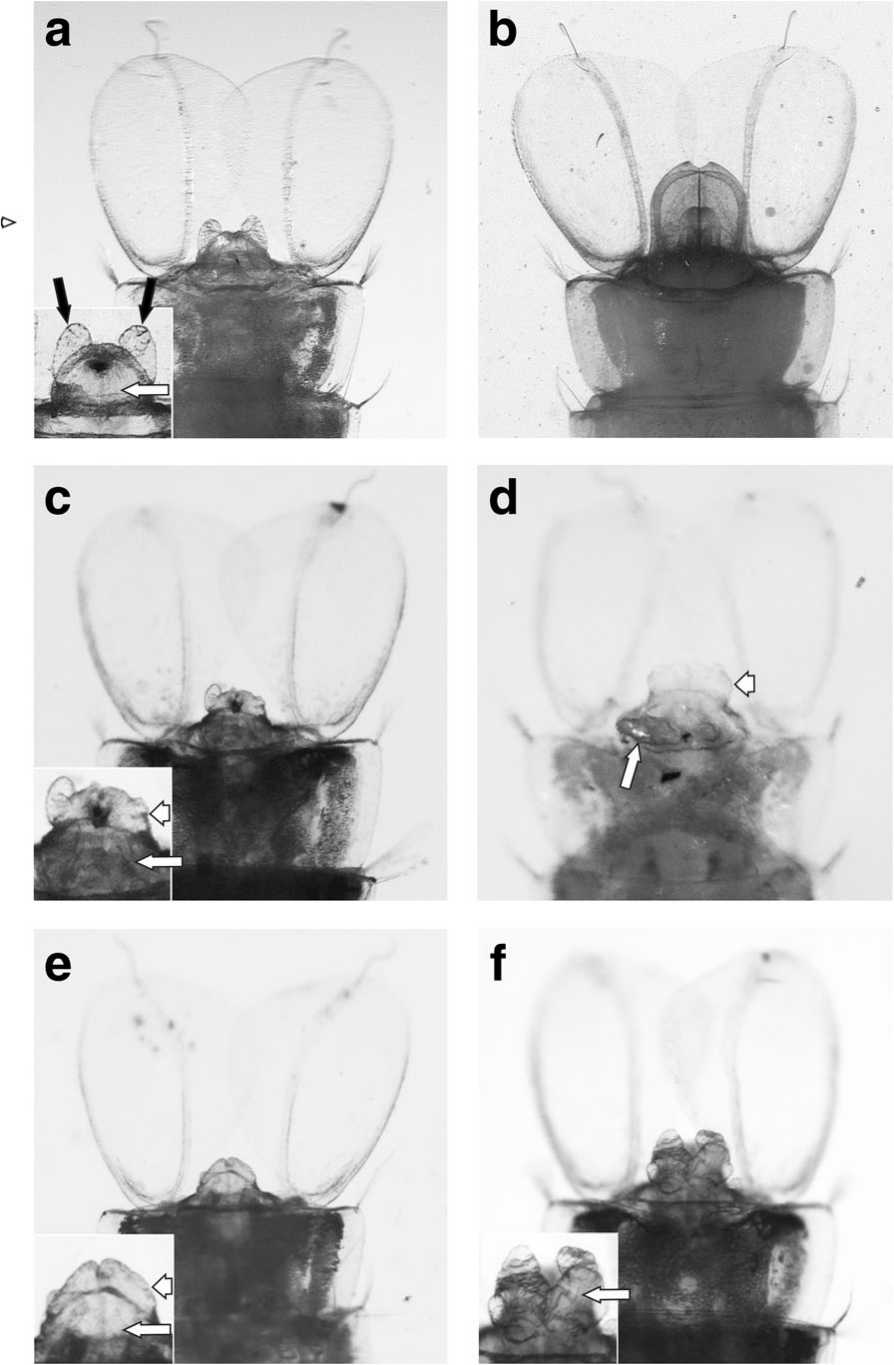
Abdominal termini of *An. gambiae* pupae, ventral aspect. **a** Wild-type female. **b** Wild-type male. **c-f** Transgenic females. Insets show enlarged genital lobe and cerci area. Black arrows indicate normally developed cerci, long white arrows indicate genital lobe and short white arrows indicate proctiger. Note underdeveloped (**c**) or missing cerci (**d-f**), tubercles and projections on the genital lobe (**d, f**), and an enlarged proctiger extending beyond the genital lobe (**c**, **d**, **e**). In wild-type female pupae (**a**) proctiger is not visible

**Fig. 3:**
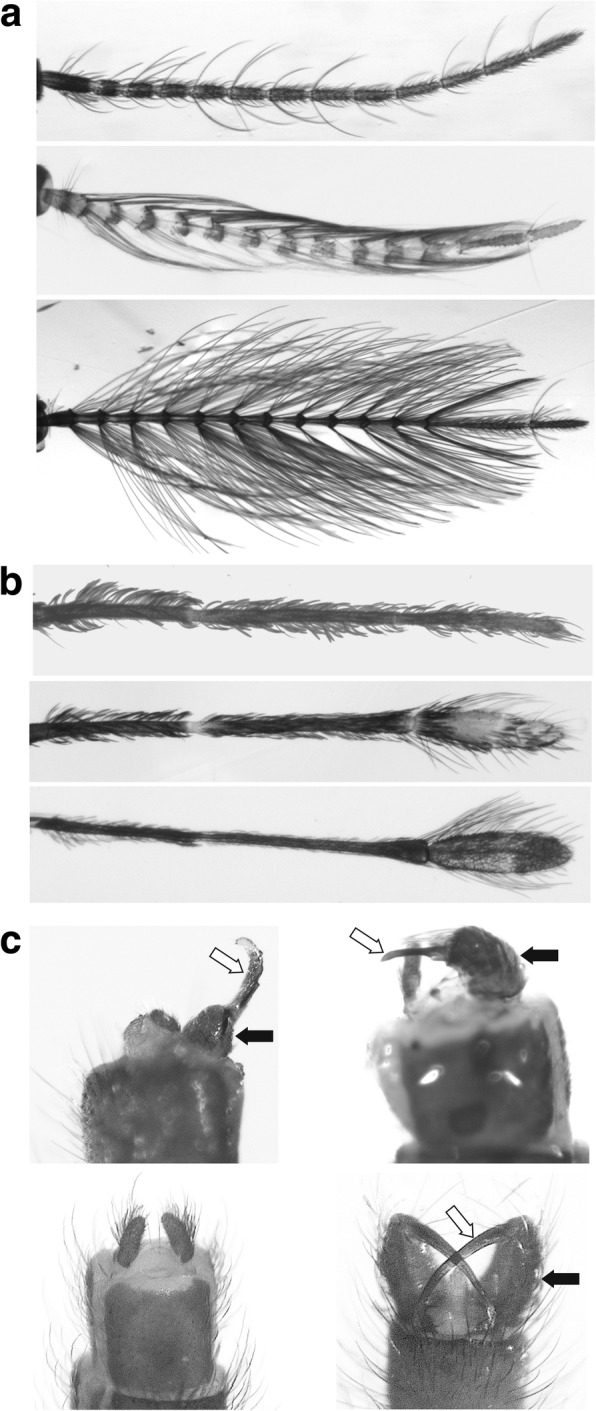
Details of *An. gambiae* adult morphology. **a** Antenna; top to bottom: wild-type female, transgenic female, wild type male. **b** Maxillary palp; top to bottom: wild-type female, transgenic female, wild type male. Antennae (**a**) and palps (**b**) oriented with distal segments on the right. **c** Abdominal termini; top: transgenic females with a partially developed gonocoxite (black arrow) and a gonostyle (white arrow); bottom left: wild-type female, bottom right: wild-type male (the gonocoxite and gonostyle indicated with arrows as in **c**)

Microscopic examination of the line 23 individuals revealed that all the transgenic female pupae showed varying degrees of intersex phenotypes. Unlike wild-type individuals (Fig. 2a), transgenic females possessed developmental defects in the terminal abdominal segments (Fig. 2c-f; end of an abdomen of a wild-type male is shown in Fig. 2b for comparison). The genital lobe was usually small, or misshapen and often bearing tubercles or longer projections directed ventrally or posteriorly. Cerci were undersized and uneven, or absent altogether, and proctiger was enlarged. Occasionally, male genitalia were partially developed. The adult females that successfully passed eclosion had male-like pilose antennae, with antennal segments IV-XIII bulbous, and two distal segments (segments XIV-XV) elongated (Fig. 3a). The last segment of the palps was distended and carrying long setae, somewhat similar to the palps in males (Fig. 3b). Moreover, some of the females possessed partially developed claspers, with clearly distinguishable gonocoxites and gonostyles (Fig. 3c).

### Altered gene expression in line 23 females

**Fig. 4:**
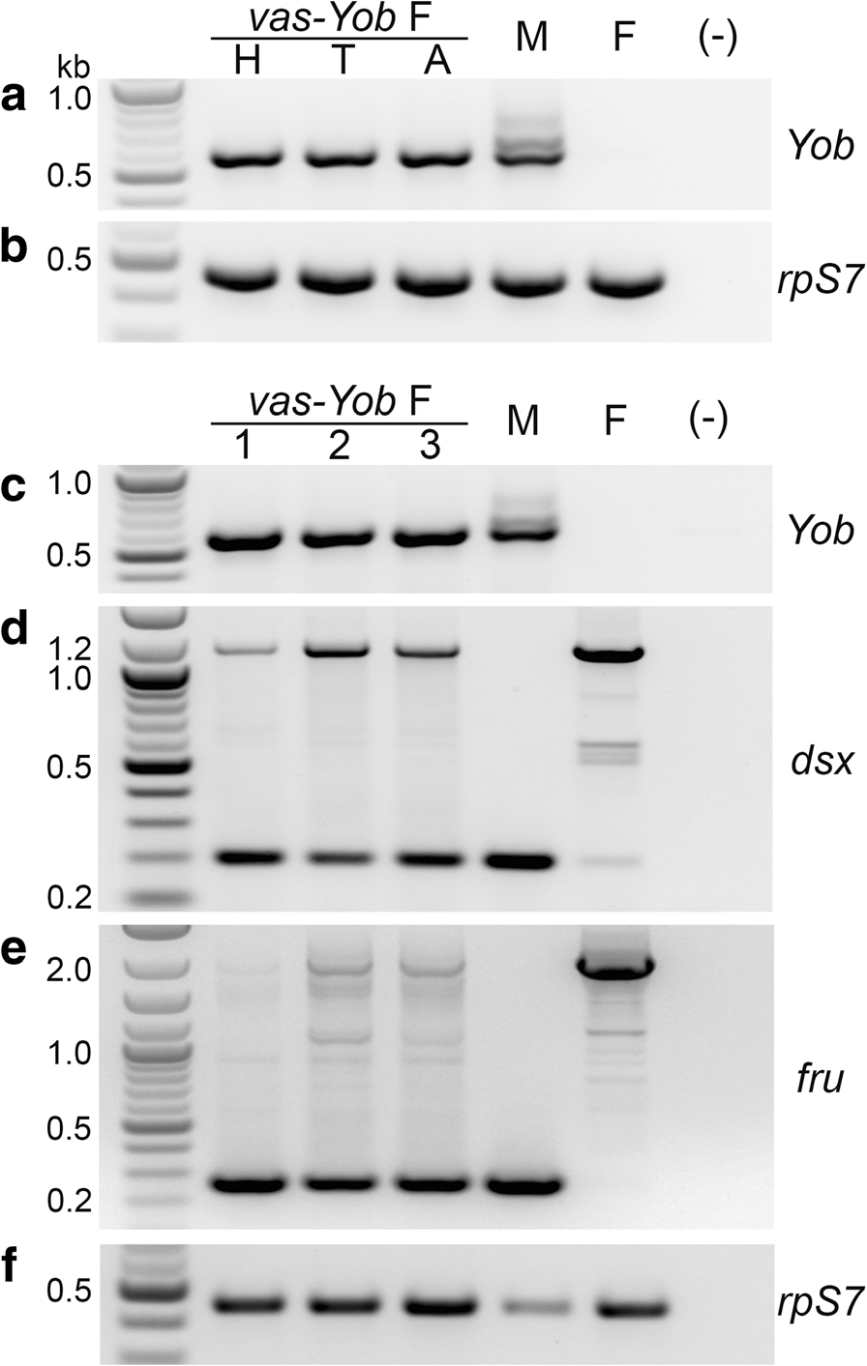
Reverse transcription (RT)-PCR analysis showing altered transcription and pre-mRNA splicing in adult females from the *An. gambiae* transgenic line 23. **a** *Yob* transcript levels in head (H), thorax (T) and abdomen (A) of a transgenic female. **b** Ribosomal protein S7 transcript levels used as a gel loading control. The same RNA samples were used in (**a**) and (**b**). **c-f** RT-PCR analysis of transcription in three randomly selected transgenic females (Lanes 1-3). **c** *Yob* transcript levels. **d** *dsx* splicing pattern. **e** *fru* splicing pattern. **f** Ribosomal protein S7 transcript levels used as a gel loading control. The same RNA samples were used in (**c-f**). In each experiment a whole body RNA sample from a wild-type adult male (M) and female (F) were used for comparison. Note additional, longer *Yob* transcript isoforms resulting from intron retention in a male; *Yob* transcripts in transgenic females originate from a cDNA clone, thus lack introns. (-) negative control

The transgenic construct used in this study carries the *Yob* coding sequence under the control of the *vas2* gene promoter, whose endogenous pattern of expression is limited to the germline [11]. In line 23, the construct is inserted on one of the two autosomes (within the first intron of the AGAP012516 gene located on an unmapped scaffold NW_045524) and *Yob* is expressed in both sexes (Fig. 4). However, this ectopic expression does not follow the *vas2* expression pattern, as indicated by comparable levels of *Yob* transcripts detected through RT-PCR in adult female heads, thoraces and abdomens (Fig. 4a; samples from other developmental stages were not analyzed).

Consistent with the role of *Yob* as the primary signal gene required for triggering the male sex determination process [3], we observed in transgenic females a very strong shift in the splicing pattern of *dsx* from the female form toward the male form (Fig. 4d). There was also a dramatic shift in splicing of the *fruitless* (*fru*) gene (Fig. 4e), which represents a branch in the sex determination pathway, and which, in *Drosophila*, is known to contribute to the differentiation of the neural circuits underlying male sexual behaviour.

## Discussion

Manipulation with transcription of the sex determination pathway genes, combined with transgenic repressible expression systems [17], may constitute a highly efficient approach to a large-scale production of males for genetic mosquito control programmes. Our study, which exploited the female killing characteristics of the primary sex determination gene *Yob* from *An. gambiae*, represents an important step towards this goal. It provides evidence that it is feasible to create *An. gambiae* transgenic lines with stable ectopic *Yob* expression, which is highly deleterious to females, but leaves males viable, fertile and apparently not affected by *Yob* overexpression.

The transgenic lines generated in this study dramatically differ in the transgene expression levels due to the chromosomal position effects of the transgene integration sites. Whereas in line 1 the expression of the DsRed marker was strongly suppressed, the marker expression in line 23 has been influenced by the presence of an unknown local enhancer or a promoter located near the transgene, which drives a high level of DsRed expression in what appear to be neurohemal organs, in addition to the eyes and the central nervous system. There was a clear correlation between the intensity of the DsRed expression and the male bias. The deficiency of females was most pronounced in line 23, which indicates that, similar to the DsRed expression, *Yob* was most strongly expressed in that line.

As a result of ectopic *Yob* expression and the presumed concomitant abnormal activation of dosage compensation in females, nearly half of the female progeny in line 23 die as embryos. However, despite carrying the same transgene, a considerable proportion of females survive to the pupal or the adult stage. Incomplete penetrance of the female embryonic lethal phenotype must result from a combination of two components: a relatively weak activity of the *vas2* promoter driving *Yob* expression in the embryo, and a considerable variability in the genetic background of the parental G3 strain used for continuous outcrossing of line 23. Segregating genetic variants in the wild-type genetic backgrounds are well known to significantly influence the phenotypic effects of mutations [18, 19]. One potential explanation for these phenomena is the existence of sequence polymorphisms in binding sites that may lead to differential binding of transcription factors, and thus affect gene expression [18]. Presence of viable females inheriting the transgenic construct in line 23 may be plausibly interpreted by such genetic background modifiers (polymorphisms) in the regulatory regions, such as in the *Yob* enhancer mentioned above, and/or other genomic loci. It is likely that certain background variants result in a decreased expression of *Yob* or downstream genes, and prevent lethal buildup of transcripts from the hyper-activated X chromosome. An alternative, not mutually exclusive mechanism may involve segregating polymorphisms that augment expression of genes causing an increased resilience to stress associated with the X chromosome hyper-transcription. Because line 23 is maintained by continuous outcrossing of the transgenic males to wild-type females (line 23 females produce no offspring), there is no selection for permissive polymorphisms that lead to survival of *Yob-*expressing females.

The viable females from line 23 carried a number of developmental defects, manifested by masculinization of head appendages and terminal abdominal segments, indicative of significantly perturbed sexual development. In all the studied females, transcripts of *dsx* and *fru*, the terminal sex determination pathway genes that control all downstream sexual developmental processes and adult male behaviour, were abnormally spliced (strong admixture of male isoforms). Lack of interest in host-seeking or blood-feeding behaviours is undoubtedly also a consequence of perturbed splicing, likely of the *fru* gene.

The economics of male mosquitoes’ production for genetic control approaches dictates that all females should be eliminated from the release generation during the embryonic stage, because the fitness and mating competitiveness of adults is highly dependent on larval density. Achieving complete female lethality driven by ectopic *Yob* expression would require use of promoters that are more active during the early zygotic stage than *vas2* used in our study. Support for this notion is given by the male-only phenotype in the transgenic lines of *An. stephensi*, in which female killing was accomplished by expression of an autosomally-integrated construct consisting of the presumed male-determining gene *Guy1* driven by its own endogenous promoter [20]. Work on suitable *An. gambiae* promoters and on transgenic constructs allowing conditional regulation of *Yob* expression (to enable generation of homozygous transgenic individuals) is currently ongoing in our laboratory.

## Conclusions

Our study demonstrates that ectopic expression of *Yob* in transgenic lines results in a recovery of viable, fertile males, and in lethality, or otherwise strongly deleterious effects, in females. Although we have not produced *An. gambiae* sexing strains per se, because a proportion of female progeny were viable, our results show potential for generation of such strains when stronger promoters are used to drive activity of *Yob* in early embryos.
